# Health supply chain system in Uganda: assessment of status and of performance of health facilities

**DOI:** 10.1186/s40545-022-00452-w

**Published:** 2022-10-05

**Authors:** Eric Lugada, Irene Ochola, Anthony Kirunda, Moses Sembatya, Sheila Mwebaze, Martin Olowo, Denis Okidi Ladwar, Henry Komakech

**Affiliations:** USAID/Strengthening Supply Chain Systems Activity, Uganda, Management Sciences for Health, Plot 15, Princess Anne Drive, Bugolobi, P. O. Box 71419, Kampala, Uganda

**Keywords:** Health supply chain, Supply chain, Status, Performance drivers, Uganda

## Abstract

**Background:**

Health supply chain systems are essential for effective and efficient healthcare system by ensuring availability of quality essential medicines and health supplies. While several interventions have been made to ensure the availability of quality essential medicines and health supplies, health facilities continue to report stockouts in Uganda.

**Objectives:**

This study aimed to assess the status and performance of the supply chain system across all levels of care in health facilities in Uganda.

**Methods:**

This was a cross-sectional study conducted in 128 public and private-not-for-profit health facilities across 48 districts in Uganda. These facilities included all levels of care from Health Centres II, III, IV, general and referral hospitals, and national referral hospitals. Data were collected using desk reviews, health facility surveys, and key informant interviews with key personnel. Stock registers were reviewed to assess the availability of a basket of essential medicines based on the essential medicines list of the Ministry of Health.

**Results:**

Less than half (42%) of health facilities had computer hardware. Most (84%) of health facilities were using a form of Logistics Management Information System with only (6%) were using the Electronic Logistics Management Information System. Just under a third (33%) of health information officers and (51%) of public health officers’ positions were filled in the health facilities. Nearly (66%) of health facilities used supply chain data to support decision-making. Most (84%) of health facilities reported stockouts of Essential Medicines and Health Supplies in the past 6 months. The main reasons for stockouts were (59%) a sudden increase in demand (40%) delivery gaps/delayed deliveries and (35%) discrepancies in orders and deliveries. Health facilities responded to stockouts through various means including (75%) redistribution (43%) purchased from a distributor, and (30%) placing emergency orders.

**Conclusions:**

The findings from this study show that the performance of health facilities in different supply chain processes and functions was defective. To improve the supply chain performance of health facilities, it is important to invest in infrastructure development, provide computer hardware and internet connection and strengthen  the capacity key personnel. This is key for ensuring full functionality of the supply chain and availability of quality medicines and health supplies to the end-user.

## Background

The health supply chain system is essential in ensuring the availability of quality Essential Medicines and Health Supplies (EMHS) and the highest possible standards of care for patients [23, 29]. Essential medicines can save lives, reduce suffering, and improve health outcomes. It is vital that the health supply chain is managed effectively to ensure the availability of EMHS and the overall goal of the health system. Effective and efficient health supply chain systems ensure access to quality and affordable EMHS throughout the healthcare system. Furthermore, the availability of medicines is an important determinant of demand, access, and utilization of health services [18, 30]. However, the limited availability of medicines and supplies is a common feature across health systems in many developing countries [25, 26]. Health workers depend on the availability of quality EMHS to deliver quality care to clients. Stockouts of medicines in health facilities discourage clients from utilizing health services [17].

Availability of EMHS in health facilities throughout the healthcare system is centred around different health supply chain processes and functions [3]. The processes and functions include forecasting, lifecycle planning, procurement, shipping, storage and issuing to patients [10]. Effectiveness of the various processes and functions has important implications for overall availability of quality EMHS across health facilities. The aim of the health supply chain system is to maintain and promote public health that guarantees the best quality of life. As such, it is essential to continuously assess the system and put in place interventions to solve challenges that may affect the performance and overall availability of EMHS across the health system.

In Uganda, the health supply chain system like the overall health system is structured at four levels to support the distribution of EMHS. The supply chain system includes several other stakeholders involved in procurement, import, wholesale, distribution, retailing and various other functions to ensure commodities reach the end users across various health facilities in the country. The structure of the system used to distribute EMHS includes national level central warehouses, district level stores, and health facility stores [15]. The structure of the health supply chain system has important implications for the performance of the health system across all levels. Previous studies have showed poor status and performance of the health supply chain system [4, 5, 8, 9, 11]. These studies highlight lack of knowledge and skills among staff, inadequate equipment and infrastructure, and poor management. All these factors contribute to poor performance and affect availability of EMHS. Despite this, few studies have assessed the status and performance of supply chain functions by health facilities. Understanding the status and performance in health facilities is important for informing the design and implementation of interventions aimed at strengthening the health supply chain system in Uganda.

This study aimed to determine the status and performance of the health supply chain system in Uganda. The study reflects the baseline situation on status and the of performance of the supply chain system in health facilities in Uganda. The study not only assesses the baseline situation but also serves to inform interventions and as a reference to evaluate the impact of interventions on the supply chain system in Uganda. In this paper, we report on the availability of EMHS in health facilities across all levels of the care system in Uganda. In addition, we report on the availability of computer and internet connectivity, Logistics Management Information System (LMIS), human resources for supply chain, regulatory and governance frameworks for supply chain management and general management of the supply chain system.

## Methods

### Study design and area

This was a facility-based cross-sectional study to assess the status, and performance of the health supply chain system in health facilities across all levels of care in Uganda. The study employed both quantitative and qualitative data collection methods. The quantitative data were collected using a health facility survey. The survey was conducted in a sample of 128 public and Private-Not-for-Profit (PNFP) facilities across all levels of care in Uganda from October to December 2020. These included all health facilities Health Centres II, III, IV General, Regional Referral, and National Referral Hospitals. Qualitative data were collected using Key Informant Interviews (KII).

### Structure of the health supply chain system in Uganda

The health supply chain (HSC) system is composed of both public and private stakeholders. The public sector HSC is coordinated by the Department of Pharmaceuticals and Natural Medicines, with the National Drug Authority (NDA) and National Medical Stores (NMS) carrying out the roles of regulation, procurement, warehousing, and distribution. The Private-Not-for-Profit sector is regulated under faith-based medical bureaux. The PNFP facilities are served by one major warehouse, joint medical stores (JMS). In addition, there are many private for-profit health facilities and pharmaceutical outlets, distributed all over the country. Private warehouses exist, mainly contracted by donors and development partners for storage of extra stock that cannot be held NMS and JMS. The pharmaceutical manufacturing sector is consistently growing, with 18 manufacturing facilities contributing 230 out of the 5,267 products in the national drug register [24].

The public sector HSC flow of products is based on two models, a pull system for health centre (HC-IVs) and above and assisted push or kit system for lower levels (HC II and HC III). Health product distribution is designed along the decentralised model with products for HC-IV and below being delivered to the office of the District Health Office (DHO) through the last-mile delivery. Not all districts have district medicines stores. Facilities report through the HMIS, which is also used to aggregate medicines orders.

### Study population and sampling

The study population included supply chain units of public and PNFP facilities across all the geographical regions of the country. In-depth interviews were conducted with the in-charge of the selected health facilities; the administrative, technical, and political leaders at the district level (District Health Office (DHO), Chief Administrative Officer and officials at NMS, JMS, Ministry of Health (MOH), and Ministry of Finance Planning and Economic Development (MOFPED).

A multi-stage sampling procedure was used to select the districts and health facilities. Four criteria were used to determine the health facilities and districts to be included in the study. These include (1) representation of all the regions; (2) cover all the National Referral (NRHs) and Regional Referral Hospitals (RRHs); (3) representation of public and PNFP health facilities; and (4) include a larger number of higher level health facilities. The Ministry of Health’s National Health Facility Master List (2018), a list of all the public and PNFP health facilities in Uganda by region and districts was used as a sampling frame.

The health facility sampling generated a total of 138 health facilities. The target number of 138 facilities was considered appropriate to have a representative sample of facilities across all levels of care. In addition, this was informed by previous studies notably the Service Availability and Reliability Assessment (SARA) survey in Uganda, which considered about 85 health facilities [20].

To determine the districts and health facilities in the study, the following steps were taken. In step 1, we considered all the districts with a National and/or Regional Referral Hospital (15 Hospitals). In step 2, the remaining 120 health facilities (excluding NRH and RRH) were split into: 50% (60 higher level health facilities) with 30 hospitals and 30 HC IV; and (60 lower level health facilities) for HC II–IV. In step 3, the ratio of public to PNFP hospitals and HC IVs were determined based on the health facility master list. Based on the ratios obtained, 30 hospitals were sub-divided into 13 public and 17 PNFP facilities. For the HC-IV, the ratio resulted in 27 public and three PNFP HC IVs. The 60 lower level facilities were further distributed into 36 HC III and 24 HC IIs. In step 4, the 13 public and 17 PNFP hospitals were randomly selected from a list of all hospitals. Likewise, the 27 public HC IVs and 3 PNFP HC IVs were randomly selected from the list of facilities in the respective category. In step 5, we identified the districts, where all sampled in step 4 health facilities selected are located. In step 6, we combined the districts in step 1 and 5 to form a final list of districts selected for the survey and to guide the selection of HC IIIs and HC IIs. This was to optimize resources, such that any district included, has at least a hospital or HC-IV to be covered, rather than having research teams travelling to a district just to cover a HC III or HC II only. In step 7, the list of HC IIIs and HC IIs in the districts identified in step 6 was used to randomly select the 36 HC IIIs and 24 HC IIs included in the assessment.

The study’s inclusion criteria for the study were the presence of a functional store and dispensing unit, supply chain staff working in a facility for 6 months or more, while interns, and clinicians were excluded from the study.

Key informants were selected using purposive sampling technique in the respective supply chain levels national, district and health facilities. The research team approached district health managers and requested for district and health facility staff who could provide information about the health supply chain system. Each potential informant identified was asked if they were able and willing to participate in the study and, if so, they were asked to give their written and informed consent. Our aim was to recruit at least one manager or administrator from each district and a health facility manager and supply chain staff from each facility. However, using snowbowl sampling technique, more interviews were interviewed using until our data became saturated and no new information emerged.

### Data collection

Quantitative data were collected using a semi-structured questionnaire. The questionnaire was adapted from tools for logistics system assessment developed by USAID/DELIVER [16]. The study questionnaire included several items including logistic management information system, competence in logistic management information system, human resources for health supply chain, availability of EMHS (stock-outs, reasons for and response to stock-outs), support supervision from the District Health Office and implementing partners. The questionnaire was pretested in six health facilities that did not form part of the sampled facilities. Based on the feedback from the pre-test, the questionnaire was modified. Aspects of the tool which were out of the intended facility’s scope/mandate (such as product selection, procurement, etc.) or which were of less significance to objectives of the study were excluded. We used Computer-Assisted Personal Interviews (CAPI) using the KOBO collect application to collect data. The KOBO collect application is a mobile-based application that facilitates data collection using mobile devices, analysis, and storage—either offline or online. Data were collected by trained research assistants. During the data collection, COVID-19 mitigation measures were used to ensure protection of research assistants and respondents.

Qualitative data were collected using an open-ended interviewer administered interview guide. The interview guide was developed for this study after reviewing different related literatures. The interview guide was prepared in English. The guide was pre-tested in health facilities not sampled for the study. The purpose of the pretest was to check the understand ability of the questionnaire, ability of the questionnaire to address the objectives of the proposed objectives and appropriateness of expressions for the context of the study areas. Based on the pretest results, minor modification was done on the contents of the questionnaire.

Data were collected by the authors and trained research assistants. All data collectors were trained for 3 days. The regarding obtaining informed consent, the objectives of the study, content of the study tools, data collection procedure, conducting interviews and data transcription. Prior to data collection, written consent was obtained from each participant after providing a brief explanation on the scope and objectives of the study. Respondents were also informed of their rights including withdrawing their participation at any time during the study and no harm would be imposed on them. All interviews were conducted in a private location of convenience to the respondent. All interviews were conducted in English. Each interview was conducted by a moderator and note taker. Interviews lasted between 60 min to one and half hours. All interviews were audio-recorded after obtaining consent from respondents. The information from all audios were transcribed in verbatim.

### Data quality management

Data collectors and supervisors were trained for 2 days with the third day used as a pre-test day. The training covered several areas including the study objectives, data collection tools, process of data collection, using the KOBO collect tool for capturing and synchronizing data, and obtaining approval and consent from study participants. A pre-test was conducted prior to beginning the data collection. The KOBO collect survey tool with validation checks was important in controlling the quality of data submitted using mobile devices. The authors, data manager and supervisors checked the data for completeness before it was uploaded to the KOBO collect server.

### Data analysis

Data collected were downloaded from KOBO collect survey tool, edited, and cleaned for consistency, missing values, using excel and exported to STATA SE 14 for analysis. Descriptive statistics were computed and summarized in tables, figures, and text with frequencies, mean, or standard deviations where appropriate. The analysis was conducted for each of the functional areas, including availability of computers and internet connection, LMIS software, competencies in LMIS, human resources for supply chain management regulatory and governance framework, technical support from the District Health Officers and implementing partners. This was conducted according to facility ownership and different levels of care. Performance in this study was defined as the extent to which activities by which the system meets end-client requirements including use and functionality of ELMIS, availability of EMHS, logistics management and inventory practices, stock outs, and capacity to deliver in a responsive manner.

Qualitative data were analysed manually using the principles of content analysis. Interviews were transcribed concurrently with data collection. Texts were read independently by the authors. Codes were developed in reference to the research questions. Codes were organized according to the conceptual themes. The authors discussed the codes and themes across all the interviews at group meetings until consensus was reached. The authors identified sections of the original transcripts and key quotes considered to be illustrative of the emerging themes that were used to guide the discussion points of the article. We employed a narrative strategy to support interpretation of the results.

## Results

### Characteristics of health facilities

A total of 128 health facilities were included in the assessment with a response rate of (93%) (Table 1). These included 91 public and 37 PNFP facilities at all levels of care from Health Centre II to National Referral Hospitals.

**Table 1:** Characteristics of health facilities

Level of care	No. of Public health facilities	No. of PNFP health facilities	Total Noh of health facilities	(%)
Health Centre II	15	9	24	18.8
Health Centre III	25	10	35	27.3
Health Centre IV	28	3	31	24.2
General Hospital	8	15	23	18.0
National Referral Hospital	2	0	2	1.6
Regional Referral Hospital	13	0	13	10.2
Total	91	37	128	100

### Use of Logistics Management Information Systems used by health facilities

**Fig. 1:**
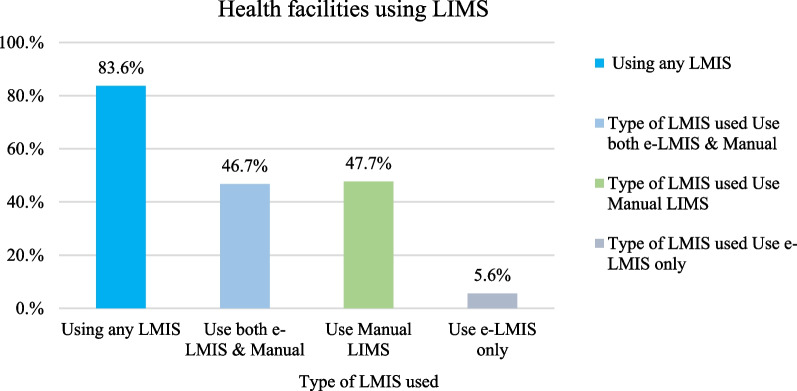
Logistics Management Information Systems used by health facilities

About (84%) of the health facilities were using some form of Logistics Management Information System (LMIS) (Fig. 1). However, only (6%) were currently using the Electronic Logistics Management Information System (ELMIS) (Fig. 1). Almost half, (47%) of health facilities were using both ELMIS and manual systems.

### Human resources for supply chain management

Overall, the position of Health Information Officer is the most filled (33.6%) across all levels of care (Table 2). Just over half (51.6%) of all public health facilities have the position of Health Information Officer filled. The position of Dispenser/Pharmacy Technician was the most filled (70%) in health facilities across all levels of care. Overall, public health facilities have supply chain positions filled compared to PNFP facilities.

**Table 2:** Supply chain positions at health facilities

	Ownership			Level of care					
	Public n = 91	PNFP n = 37	HC II n = 24	HC III n = 35	HC IV n = 31	GH n = 23	RRH n = 15	NRH n = 14	Overall
Inventory management officers	21 (23.1%)	10 (27.0%)	4 (16.7%)	4 (11.4%)	7 (22.6%)	5 (21.7%)	3 (20.0%)	1 (7.1%)	18.8%
Assistant Inventory Management Officer	26 (28.6%)	5 (13.5%)	3 (12.5%)	3 (8.6%)	12 (38.7%)	7 (30.4%)	7 (46.7%)	1 (7.1%)	25.8%
Health Information Officer	47 (51.6%)	13 (35.1%)	3 (12.5%)	13 (37.1%)	18 (58.1%)	4 (17.4%)	4 (26.7%)	1 (7.1%)	33.6%
Pharmacists	14 (15.4%)	4 (10.8%)	1 (4.2%)	1 (2.9%)	2 (6.5%)	6 (26.1%)	6 (40.0%)	1 (7.1%)	14.1%
Dispenser/pharmacy technician	44 (48.4%)	26 (70.3%)	1 (4.2%)	4 (11.4%)	8 (25.8%)	7 (30.4%)	1 (6.7%)	1 (7.1%)	17.2%
Driver	20 (20.0%)	17 (45.9%)	1 (4.2%)	2 (5.7%)	4 (12.9%)	5 (21.7%)	2 (13.3%)	1 (7.1%)	11.7%

Key informants identified staff shortage as a challenge affecting supply chain function and processes in the district and health facilities. Staff shortage was identified as a reason affecting supply chain performance in health facilities across all levels of care. One respondent had this to say:

*“Of course, Human Resource is important. It determines everything that has to be moved by humans and we are a little bit understaffed. This also is reflecting on the performance of supply chain department in in the district and health facilities. The processes and functions are not effective because of inadequate supply chain staff. If we had a bit more employment, I think work would be better.” (District Health Officer, #1 Male)*

Functionality of ELMIS in health facilities varied across nine indicators (Table 3). Overall, less than half (42.2%) of all health facilities have functional hardware to support supply chain functions. Most (86.7%) of Regional Referral Hospitals have functional hardware and ELMIS software to support supply chain functions. About (76.6%) of health facilities have staff trained in LMIS stock management and record keeping. Only (4.2%) of HC IIs had functional hardware and ELMIS software to support supply chain functions.

**Table 3:** Functionality of ELMIS in health facilities

ELMIS functionality	Ownership		Level of care						
	Public n = 91	PNFP n = 37	HC II n = 24	HC III n = 35	HC IV n = 31	GH n = 23	RH n = 15	NRH n = 14	Overall
Availability of functional hardware	38 (41.8%)	17 (45.9%)	1 (4.2%)	3 (8.6%)	16 (51.6%)	20 (87.0%)	13 (86.7%)	1 (7.1%)	42.2%
Availability of functional ELMIS software	34 (30.9%)	17 (45.9%)	1 (4.2%)	4 (11.4%)	14 (45.2%)	18 (78.3%)	13 (86.7%)	1 (7.1%)	39.8%
Internet connection to support ELMIS	28 (25.5%)	15 (40.5%)	0 (0.0%)	3 (8.6%)	11 (35.5%)	16 (69.6%)	13 (86.7%)	0 (0.0%)	33.6%
Use ELMIS to submit reports to NMS/JMS/MAUL	28 (25.5)	13 (35.1%)	1 (4.2%)	3 (8.6%)	13 (41.9%)	14 (60.9%)	10 (66.7%)	0 (0.0%)	32.0%
USE ELMIS to monitor and evaluate performance	26 (23.7%)	14 (37.8%)	11 (45.8%)	16 (45.7%)	10 (32.3%)	6 (26.1%)	7 (46.7%)	1 (71%)	39.8%
Staff trained in the use of ELMIS	42 (38.2)	15 (40.5%)	9 (37.5%)	16 (45.7%)	5 (16.1%)	4 (17.4%)	0. (0.0%)	0. (0.0%)	26.6%
ELMIS is usable and acceptable	31 (28.2%)	15 (40.5%)	8 (33.3%)	11 (31.4%)	12 (38.7%)	4 (17.4%)	1 (6.7%)	1 (7.1%)	28.9%
Staff trained in LMIS stock management and record keeping	68 (61.9%)	30 (81.1%)	12 (50.0%)	21 (60.0%)	27 (87.1%)	23 (100%)	13 (86.7%)	2 (13.3%)	76.6%

Key informants explained that access to internet remains a big challenge to the use and functionality of ELMIS in health facilities. One respondent has this to say:

*“We have computers in the health facility and even personal ones. But having good internet is a big challenge for many health workers. Internet connection is not sufficient because we have to improvise; it is me who pays for my data to do official work in this health facility. This makes use of the ELMIS and all the functions difficult for us.” (Pharmacist #2, Male)).*

### Logistics management practices in health facilities

All (100%) of public and (97%) of private health facilities separated damaged/expired medical products from inventory and stock records (Table 4). Overall (91%) of public and (92%) of private health facilities regularly monitored stock status of EMHS. Most (87.5%) of health facilities conduct forecasting of health commodities. Almost two-thirds (65.6%) of health facilities use facility supply chain data to support decision making.

**Table 4:** Logistics management practices in health facilities

Indicator	Facility ownership			Level of care						
	Public n = 91	PNFP n = 37	HC II n = 23		HC III n = 31	HC IV n = 31	GH n = 23		RRH n = 15	Overall (%)
Using facility SC data to support decision making	59 (64.8%)	25 (67.6%)	14 (58.3%)		20 (57.1%)	19 (58.1%)	18 (78.3%)		2 (14.3%)	65.6
Forecasting health commodity requirements	81 (89.0)	32 (86.5%)	19 (79.2%)		29 (82.9%)	30 (96.8%)	20 (87.0%)		2 (14.3%)	87.5
Undertaking regular EMHS stock status	91 (100%)	34 (91.9%)	23 (95.8%)		33 (94.3%)	31 (100%)	23 (100%)		2 (14.3%)	97.7
Separate damaged/expired products	91 (100%)	36 (97.3%)	24 (100%)		34 (97.1%)	31 (100%)	23 (100%)		2 (14.3%)	99.2
Commodities on the EMHS list used	53 (58.2%)	22 (59.5%)	11 (45.8%)		18 (51.4%)	25 (80.6%)	14 (60.9%)		0 (0.0%)	58.6
Quality assurance inspection of products	83 (91.2%)	36 (97.3)	21 (87.5%)		33 (94.3%)	28 (90.3%)	23 (100%)		2 (14.3%)	93.0

### Stock-outs of EMHS by level of care

Most (83.6%) public health facilities reported stock-outs of one or more EHMS in the past 6 months (Table 5). Stock-out were lower at HC IIs compared to higher level facilities. By type of facility ownership, more public health facilities reported stock outs of EMHS compared to PNFP facilities.

**Table 5:** Stock-out levels by level of care and type of ownership

	Ownership		Level of care						
	Public (n = 91)	PNFP (n = 37)	HC II (n = 24)	HC III (n = 35)	HC IV (n = 31)	GH (n = 23)	RH (n = 15)	NRH	Overall
Stock-out in last 1 month only	76 (83.5%)	37 (100%)	15 (62.5%)	13 (37.1%)	10 (32.3%)	6 (26.1%)	2 (13.3%)	0 (0.0%)	35.9%
Stock-out in last 3 months only	16 (17.6%)	21 (56.8%)	10 (41.7%)	7 (20.0%)	7) (22.6%)	2 (8.7%)	1 (6.7%)	0 (0.0%)	21.1%
Stock-out over the last 1 to 6 months	4 (4.4%)	17 (45.9%)	9 (37.5%)	4 (11.4%)	5 (16.1%)	2 (8.7%)	1 (6.7%)	0 (0.0%)	16.4%

Managers and health service providers indicated that generally, stock outs happen on a regular basis. Informants observed that availability of drugs is an important determinant of services provided by health facilities. One key informant has this to say:

*“Drug stock outs remain a challenge in the district and in many health facilities. This happens frequently and has a bad effect on service availability in health facilities as it determines whether we are actually working. If drugs are not there in health facilities, it actually means that we are not working. There is irregular supply of medicines and other commodities.” (District Health Officer #3, Male)*

### Reasons for stock-out of EMHS in health facilities

The main reasons for stock out of EHMS include (59%) sudden increase in demand for some medicines, (40%) delivery gaps/delayed deliveries, and (35%) discrepancies in orders and deliveries (Table 6). The main reasons for stock outs seem consistent across all levels of care and facility ownership.

**Table 6:** Reasons for EMHS stock-out at Health facility level

Reasons for stock-outs	Facility ownership		Level of care					
	Public n = 91	PNFP n = 37	HC II n = 24	HC III n = 35	HC IV n = 31	GH n = 23	RRH n = 15	Overall
Discrepancies in orders and deliveries	22 (24.1%)	9 (24.3%)	4 (16.7%)	7 (20.0%)	11 (35.5%)	6 (26.1%)	3 (20.0%)	31 (24.2%)
EMHS shortages	33 (36.3%)	10 (27.0%)	6 (25.0%)	13 (37.1%)	9 (29.0%)	9 (39.1%)	5 (33.3%)	43 (33.6%)
Errors in ordering/requesting EMHS	6 (6.6%)	3 (8.1%)	0 (0.0%)	1 (2.9%)	3 (9.7%)	2 (8.7%)	2 (13.3%)	8 (6.3%)
Delivery gaps (delayed deliveries)	30 (33.0%)	13 (35.1%)	5 (20.8%)	9 (25.7%)	13 (41.9%)	9 (39.1%)	5 (33.3%)	43 (33.6%)
Sudden increase in demand for some EMHS	42 (46.2%)	16 (43.2%)	10 (41.7%)	18 (51.4%)	12 (38.7%)	12 (52.2%)	5 (33.3%)	58 (45.3%)
Ordering system failure	5 (5.5%)	4 (10.8%)	1 (4.2%)	4 (11.4%)	0 (0.0%)	3 (13.0%)	1 (6.7%)	9 (7.0%)
Limited capacity in quantification, and forecasting	12 (13.2%)	2 (5.4%)	3 (12.5%)	4 (11.4%)	3 (9.7%)	1 (4.3%)	3 (20.0%)	14 (10.9%)
Lack of incentive to maintain stock	1 (11.0%)	1 (2.7%)	0 (0.0%)	1 (2.9%)	1 (3.2%)	0 (0.0%)	0 (0.0%)	2 (1.6%)

Key informants mentioned that stock outs were a result of several reasons. Delays in the delivery of medicines was highlighted as one of the main reasons for stock outs by respondents. One key informant has this to say:

*“Sometimes National Medical Stores can delay delivering medicines, that’s an operational constraint. So, you will have no medicines because of a late delivery also if anything is missing at NMS it will affect your facility, it’s not like we have a choice if somethings missing we have another source so if something is missing at our soul supplier we are finished.” (Pharmacist, Male)*

### Responses to EMHS stock-outs by health factions

Most (75.9%) public health facilities rely on redistribution of EMHS from other facilities to address stock-outs (Table 7). About (43.2%) of PNFP facilities bought EMHS from a distributor/pharmacy. Nearly (29.7%) of public health facilities placed emergency orders in case of stock-outs.

**Table 7:** Response to stock-outs by health facilities across all levels of care

	Facility ownership		Level of care					
Response	Public n = 91	PNFP n = 37	HC II n = 24	HC III n = 35	HC IV n = 31	GH n = 23	RRH n = 15	NRH n = 5
Redistribution from other facilities	60 (65.9%)	17 (45.9%)	9 (37.5%)	24 (68.6%)	17 (54.8%)	16 (69.9%)	9 (60.0%)	2 (14.3%)
Placed an emergency order	27 (29.7)	10 (27.0%)	1 (4.2%)	10 (28.6%)	9 (29.0%)	10 (43.5%)	6 (40.0%)	1 (7.1%)
Bought from a distributor/Pharmacy	13 (14.3%)	16 (43.2%)	3 (12.5%)	8 (22.9%)	6 (19.4%)	9 (39.1%)	2 (13.3%)	1 (7.1%)
Did nothing	5 (5.5%)	1 (2.7%)	1 (4.2%)	2 (5.7%)	2 (6.5%)	1 (4.3%)	0 (0.0%)	0 (0.0%)
Others (donations, substitutions, refer)	11 (12.1%)	3 (8.1%)	2 (8.3%)	2 (5.7%)	6 (19.4%)	2 (8.7%)	1 (6.7%)	1 (7.1%)

Both managers and health facility staff indicated that in the event of stock-outs they are forced to resort to alternative means of accessing drugs to ensure that health services are not interrupted. One respondent had this to say:

*“When we experience a stock-out, we first of all seek advice from the DHO. We discuss and agree on how to manage the stock out. For example, with the advice and coordination of the DHO, we sometime exchange or get drugs from other health facilities that have more than adequate or unused stocks. (Health Facility Manager, #2, Male)*

## Discussion

The study aimed to examine the status and performance of the health supply chain system in health facilities in Uganda. The findings suggests that the health supply chain system is performing sub-optimally in several processes and functions across all levels of care. This is demonstrated by inadequate qualified human resources for supply chain, weak infrastructure, and systems to support health management information systems, and persistent stockouts of EMHS across all levels of care in the country.

The results of this study show that availability of EMHS varied according to facility ownership and level of care. Recent studies have shown different levels of availability of EMHS across health facilities in Uganda. A study by [2] showed higher levels of availability of essential medicines at across all levels of care than those observed in this study. However, the results are from a sample of 23 health facilities. In many low- and middle-income countries, the availability of Essential Medicines in public health facilities ranges between 17.9% and 87.1% [31]. The findings also show that most health facilities experience stock-outs of EMHS in the past 6 months. Overall, stock-outs were lower in lower level health facilities compared to higher level facilities. This could be due to patients by-passing lower level facilities in preference for higher level facilities, where the quality of care is perceived to be better. A survey in Uganda showed higher, 90% stock outs of EMHS in all health facilities across all levels of care in Uganda [15]. High levels of stock-outs observed in this study can be attributed to low adherence to supply plans and stock card accuracy in health facilities.

The findings also show that while majority of health facilities were using a form of LMIS, few were exclusively using the ELMIS. This hinders data collection, analysis and visibility that is key to informing decision making at managerial and policy levels. Adoption and continuous use of E-health technology applications, such as the ELMIS internet-based Health Information Systems [6], Electronic Medical Records (EMR) are essential for improving quality of healthcare delivery, increasing patient safety, and improving efficiency [1, 27]. A systematic and functional supply chain ensures availability of EMHS at the point of need. However, this requires sustainable and effective LMIS that makes data available to service providers and managers to enable evidence-based decision making and supply planning for EMHS.

Our findings show a general shortage of supply chain positions in health facilities across all levels of care. Except for RRHs, most health facilities did not have supply chain positions filled. Lower level health facilities had the least number of supply chain positions filled. In these facilities, staff often doubled as supply chain staff in addition to their key roles and responsibilities in health service delivery. These findings indicate that most health facilities were running their activities without dedicated supply chain staff. This finding supports previous studies in Ethiopia and Nigeria which showed that supply chain management was made worse by lack of trained staff [12, 14]. Globally, the health workforce remains low with low and middle income countries having the lowest number of pharmacist [7]. The availability of medicines is determined by several factors. However, there is growing recognition of the need to address the challenges including shortage of human resources for health supply chain [28]. To address these challenges, governments and partners require comprehensive and reliable data on human resources for supply chain to enable tailored policies and interventions.

The present findings show high levels of performance in a few areas supply chain practices across all levels of care. This was somewhat unexpected given inadequacies observed in other critical supply chain system including inadequate infrastructure and equipment, and lack of trained staff. A possible explanation for these results may be increased management support supervision for supply chain staff. The findings contrast with other studies [4, 5, 19] that reported weakness in the performance of various supply chain functions in health facilities. Effective supply chain management practices can have a positive impact on the performance of health facilities. To strengthen health supply chain management practices in health facilities, the Ministry of Health, District Health Office, and partners need to strengthen oversight and improve management efficiency of supply chain staff across all levels of care.

Our study identified several gaps and shortfalls in resources including human resources, equipment and software that are essential for functionality of the health supply chain system. These inadequacies have a negative effect on responsiveness, resilience, and service optimisation [4, 5]. The challenges of lack of working aids, equipment, administrative resources, and inadequate funding can be a source of frustration to supply chain staff with severe implications for quality of services for end users. These findings are consistent with other studies [13, 22]. Similarly, the findings observed that the resource gaps are compounded by inadequate skilled personnel to support supply chain functions across all levels of care. While staff took on supply chain functions in addition to their key roles and responsibilities in health facilities, this was only temporary and contributed to overall poor performance in priority areas.

This study has provided insights into the status and performance of the health supply chain system in health facilities in Uganda. The finding of inadequate infrastructure, computer equipment and information systems in health facilities is important as the governments prioritises the digitization of health service delivery in the country [21]. This finding may help inform the design and implementation of interventions aimed at strengthening infrastructure development and the provision of computers and internet. This could lead to increased efficiency and performance, streamlined processes and functions, enhanced visibility, planning and overall greater access to quality EMHS for end-users. Furthermore, the findings could be used to empower district health managers and health service providers in decision making and management of supply chain system. This is essential for decentralization in which the districts and health facilities take up greater responsibilities for the performance of the health facilities in various supply chain processes and functions.

The strength of this study is it included both quantitative and qualitative methods to supplement this study. This study has several limitations to consider as well. First, while the sample of facilities was drawn from across the country and all levels of care, the findings may not be generalizable to ownership of facilities, levels of care and districts. The qualitative component of the study focused on experiences of district and health facility personnel involved in the health supply chain system. The perspectives of other stakeholders (private sector, communities) are absent. These limitations should be considered for future studies assessing the health supply chain in Uganda.

## Conclusions

This study assessed the status and performance of the health supply chain system in health facilities in Uganda. The study found out that less than half of health facilities had hardware to support electronic logistics management. Most facilities with computer hardware, had one or more ELMIS. Most positions were not filled in all health facilities affecting the performance of supply chain functions and processes. Most facilities were stocked out of one or more EMHS in the past 6 months prior to the study. A common challenge in health facilities across all levels of care is inadequate training on key supply chain functions across all levels of care. To improve access to EMHS, it is critical to ensure availability of computers and internet connectivity to ensure the use of ELMIS to support key supply chain functions in health facilities. It is important to improve staffing levels, and on the job training and mentorship opportunities to improve knowledge and skills of staff in health facilities across all levels of care.

## Data Availability

The data sets generated and/or used for the analysis for the current study can be made available from the corresponding author on a reasonable request.

## Abbreviations

CAO: Chief Administrative Officer
DHO: District Health Officer
ELMIS: Electronic Logistics Management Information System
HC: Health Centre
JMS: Joint Medical Stores
LMIS: Logistics Management Information System
LMIS: Logistic Management Information System
MoFPED: Ministry of Finance Planning and Economic Development
MoH: Ministry of Health
NMS: National Medical Store
NRHs: National Referral Hospitals
PNFP: Private-Not-For-Profit
RRHs: Regional Referral Hospitals
